# Changing the Route of Hysterectomy into a Minimal Invasive Approach

**DOI:** 10.1155/2013/249357

**Published:** 2013-05-21

**Authors:** Christian Hoyer-Sorensen, Sigurd Hortemo, Marit Lieng

**Affiliations:** ^1^Department of Obstetrics and Gynecology, Sorlandet Hospital, Serviceboks 416, 4604 Kristiansand, Norway; ^2^Department of Gynecology, Oslo University Hospital, Postboks 4956 Nydalen, 0424 Oslo, Norway; ^3^Institute of Clinical Medicine, Oslo University Hospital, Postboks 1171 Blindern, 0318 Oslo, Norway

## Abstract

*Objective*. To describe the route of hysterectomy in a county hospital and evaluate the shift towards a minimal invasive approach. *Design*. Retrospective cohort study. *Setting*. A county hospital in Norway. *Population*. All women were scheduled for hysterectomy. *Methods*. Audit the route of hysterectomy in the period 2004–2012. Analyze the outcome of total laparoscopic hysterectomies. *Main Outcome Measures*. Complications after total laparoscopic hysterectomy. *Results*. A shift towards a minimal invasive approach has been achieved during the study period. In 2012 only 17.4% of the hysterectomies were performed abdominally, compared to yearly percentages of above 50% in the period 2004–2009. Laparoscopic supracervical hysterectomy was introduced in 2003, but the percentage of abdominal hysterectomy remained above 50% until total laparoscopic hysterectomy was introduced in 2010. Since the introduction of total laparoscopic hysterectomy in April 2010, 58 procedures have been performed. There have been no major complications. Two vaginal vault hematomas and one case of urinary tract infection were reported. *Conclusions*. It is possible for a county hospital to alter their praxis and perform mini-invasive hysterectomies, but it requires dedicated gynecologists. This change to an advanced procedure like total laparoscopic hysterectomy could be achieved without patients suffering from major complications.

## 1. Introduction

There is convincing evidence that abdominal hysterectomy is associated with a less favourable outcome compared with a vaginal or laparoscopic approach. When an abdominal approach is chosen, the woman will experience reduced quality of life, a longer time before she returns to normal activity, and a longer hospital stay [1]. The risk of complications such as wound infections is higher and the cosmetic result after abdominal hysterectomy is less favourable. Despite this, abdominal hysterectomy is still very common. In a cross-section analysis of 518 828 hysterectomies in the United States from 2005, 64% of the hysterectomies were performed abdominally whereas 14% were performed laparoscopically and 22% vaginally [2]. In the national guidelines for Norwegian gynecologists published by the Norwegian Gynecological Association, no route or approach of hysterectomy is recommended. There are differences in how hysterectomies are managed in different hospitals throughout the country [3]. In an audit evaluating the route of hysterectomy in all Norwegian hospitals in women suffering from abnormal uterine bleeding and/or leiomyoma, significant variations in the percentage of abdominal hysterectomies between the hospitals were found [4]. A reduction of the overall percentage of abdominal hysterectomies from 75% in 2003 to 62% in 2006 was found. In general, hospitals with low patient volumes tended to perform more abdominal hysterectomies compared to larger hospitals. In some small hospitals, the percentage of abdominal hysterectomy was 100%, whereas in the largest hospital, the percentage of abdominal hysterectomy performed by laparotomy was about 20%. This indicates that techniques for minimal invasive hysterectomies have not yet been adapted by most gynecologists in smaller departments, although the advantages and technique of such procedures have been frequently reported and described by colleagues from larger hospitals during the last decade. 

Our hospital is a county hospital in Norway, where approximately 120 hysterectomies are performed each year. Our main route of hysterectomy has historically been abdominally. In order to perform less laparotomies during hysterectomy, laparoscopic supracervical hysterectomy was introduced in 2003. Despite this, the percentage of abdominal hysterectomy did not decrease. The percentage of abdominal hysterectomy remained above 50% until 2009 whereas a slight decrease in the percentage of vaginal hysterectomy from 30% to about 20% was seen. 

In order to reduce the number of abdominal hysterectomies, total laparoscopic hysterectomy was then introduced in April 2010 resulting in a shift towards a laparoscopic approach. The hospital policy was altered and laparoscopic hysterectomy, total or supracervical, was chosen to be the primary route of hysterectomy. In 2012 the percentage of hysterectomy performed laparoscopically was 69.8% whereas the percentage of vaginal and abdominal hysterectomy had declined to 12.8% and 17.4%, respectively. 

The aim of this study is to present how a shift towards minimal invasive hysterectomy was achieved in a relatively small gynecological department and present the results of the first 58 total laparoscopic hysterectomies. 

## 2. Material and Methods

Laparoscopic supracervical hysterectomy (LSH) was introduced in 2003. An experienced laparoscopic surgeon was invited to the hospital and he participated in the first procedures. The procedure was introduced to some consultants who performed all LSH procedures at our hospital for the first five years. The other consultants continued to perform either vaginal or abdominal hysterectomy. 

Prior to the first total laparoscopic hysterectomy (TLH), excessive laparoscopic suture training was performed at a minimal access therapy training unit (MATTU) where proper suture skills were learned from experienced laparoscopic surgeons. The first three procedures were also performed together with an experienced laparoscopic surgeon making “hands-on” learning possible. The two first authors performed further surgery, both with large experience in laparoscopy on an intermediate level including laparoscopic supracervical hysterectomy, salpingooophorectomy, cystectomy, and laparoscopic treatment of ectopic pregnancies and moderate endometriosis as well as single-port laparoscopy.

Supracervical hysterectomy is the preferred mode of hysterectomy in our unit. That policy was continued during the shift towards laparoscopic hysterectomies. Total laparoscopic hysterectomy was performed in case of atypical endometrial hyperplasia and endometrial cancer, previous cervical intraepithelial neoplasia, and on patient request. Only FIGO stage 1 endometrial cancer is treated in our unit, as more severe stages are referred to a university hospital for treatment according to national standards. 

LSH was introduced to the other consultants in 2012 making the department less vulnerable to doctors' accessibility. If there was any preoperative uncertainty whether a planned hysterectomy could be performed by a laparoscopic approach, the case was discussed with the consultants with the largest laparoscopic experience. By placing a focus on minimal invasive hysterectomy, the threshold for performing an abdominal hysterectomy was increased. In case of a prolapsed uterus where a hysterectomy was indicated, a vaginal approach was still preferred. 

### 2.1. Surgical Technique

Preoperatively, all women were given paracetamol 1.5 grams, diclofenac 100 mg, and oxycodone 10 mg as a single dose oral analgesics. Under general anaesthesia and endotracheal intubation the patients were positioned in the dorsal lithotomy position with both legs supported in Allen stirrups and their arms resting alongside the body. An orogastric tube was placed to decompress the stomach, a Foley catheter was placed in the bladder, and 5 mL of 0.5% bupivacaine hydrochloride (Marcain) was injected as local anaesthetic before an incision was made in the umbilicus. Abdominal access was gained by using an open Hasson technique. A suture was placed in the fascia with Polysorb 0. Carbon dioxide was insufflated through this port with a pressure set at 12 mm Hg. 5 mL of 0.5% bupivacaine hydrochloride (Marcain) was injected at each of the places for three additional ports, Versaport Bladeless Trocars (Covidien, Norwalk Connecticut, USA), placed in the lower right and left quadrant and in the midline, approximately 2 cm above the symphysis. 

Prior to the total laparoscopic hysterectomies, a uterus manipulator (Colpo-Probe Vaginal Fornix Delineator) was installed in the vagina. The bladder peritoneum was opened with a Harmonic scalpel, and the bladder was pushed down over the uterus manipulator. The round ligament was divided with the Harmonic scalpel and the peritoneum was opened downwards towards the bladder. The suspensory ligament of the ovaries was divided if the adnexa were removed; otherwise the uteroovarian ligament and the proximal tube were divided. The uterine artery was coagulated with bipolar coagulation and divided with the Harmonic scalpel. A circumcision of the cervix was performed with a monopolar hook on cutting current. The uterus was removed through the vagina and the vaginal vault was sutured with three laparoscopic sutures Polysorb 0, extracorporeal suturing. 

For the laparoscopic supracervical hysterectomies, bipolar current and cold scissors were used to divide the uterus from its attachments including the uterine artery. The amputation was performed using a monopolar lap loop. Morcellation was performed through the lower left quadrant using different reusable morcellators (Gynecare Morcellex, Ethicon and Wisap, Wisap Medical Technology GmbH) and a single use morcellator (Lina Xcise, Lina Medical). 

For both procedures the fascia at the accessory ports was sutured with Polysorb 0 using the Endoclose hook (Covidien, Norwalk Connecticut, USA) if the incisions were larger than 10 mm. The fascia in the umbilicus was closed with the earlier placed suture and the skin was closed using abrupt intracutaneous Caprosyn 3-0 sutures and/or Steristrips. 

### 2.2. Data Collection Tools

Demographics and clinical data were collected from the patient's medical record after the total laparoscopic hysterectomies and a retrospective analysis was performed. Variables include BMI, parity, indication for hysterectomy, complications, and conversion to laparotomy. To evaluate possible risk factors for complications, a registration of previous abdominal surgery, intra-abdominal adhesions, and previous conisation due to cervical intraepithelial neoplasia (CIN) were also performed.

The data of complications was collected at least three months after surgery to ensure inclusion of possible late complications related to the performed surgery. 

## 3. Results

**Figure 1:**
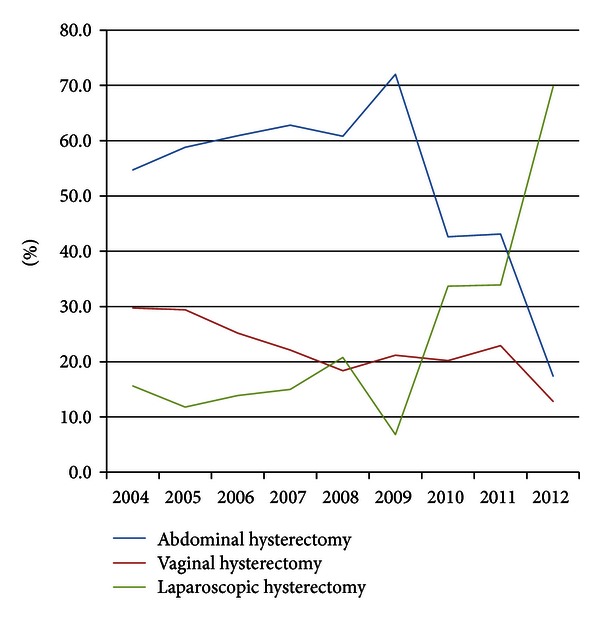
Route of hysterectomy.

The percentage of abdominal hysterectomy remained high (50–70%) until total laparoscopic hysterectomy was introduced in 2010 (Figure 1). 

 From 2010, a significant decline in the percentage of abdominal hysterectomies occurred, resulting in only 17.4% abdominal hysterectomies in 2012. The percentage of laparoscopic supracervical hysterectomy varied from 6.8% to 20.8% in the period from 2004 to 2009. In 2010 the laparoscopic group included both LSH and TLH resulting in a remarkable increase in the total number of laparoscopic hysterectomies to 69.8% in 2012. Parallel with the increase in laparoscopic hysterectomies, a decrease in vaginal hysterectomies from 29.4% to 12.8% was observed.

A total of 58 TLH procedures were performed from April 2010 to December 2012. The patient's characteristics are listed in Table 1. 

The majority of these procedures were performed on benign indications, where abnormal vaginal bleeding was the most common indication (Table 1). FIGO stage 1 endometrial cancer counted for 32.6% of the TLH performed. This group consists of older, postmenopausal women often with challenging comorbidity. The overall BMI was 27.0 (SD 6.4) whereas a BMI of 31.6 (SD 7.6) was found in women with endometrial cancer. 

No major complications such as major bleeding, bowel or urinary tract injury or vaginal cuff dehiscence occurred. Furthermore, no procedures were converted to laparotomy. There were two cases of vaginal vault hematoma. The first patient was a 40-year-old woman who had a TLH performed due to uterine leiomyoma. During the operation, excision of endometriosis on the pelvic sidewall was performed. She was readmitted six weeks after the operation with abdominal pain that had occurred after sexual intercourse. She had also experienced increased vaginal discharge. A vaginal ultrasound was performed showing a 3 × 7 cm vaginal vault hematoma. She was treated with antibiotics because of a possible infection. The hematoma resolved spontaneously. 

The second case of a possible vaginal vault hematoma occurred in a 46-year-old woman who underwent TLH because of menorrhagia. Four weeks after the operation she was admitted due to a vaginal bleeding. A transvaginal ultrasound examination showed a possible vaginal vault hematoma. She was observed for one day. The following day, a vaginal ultrasound performed by a consultant gynecologist showed no sign of a hematoma. The patient was discharged in good health.

One woman experienced a urinary tract infection together with a superficial wound infection at the suprapubic trocar incision site. Her general practitioner treated her with antibiotics and she recovered perfectly. 

## 4. Discussion

We have described a successful change from abdominal to minimal invasive hysterectomy in a relatively small county hospital. A total change of department policy, dedicated gynecologists, and competence to perform both supracervical and total laparoscopic hysterectomies seemed necessary to achieve a shift from abdominal to laparoscopic hysterectomies.

Despite the recommendations in the literature, a large amount of the hysterectomies in many western countries is still performed abdominally. Gynecologists are aware of the recommendations, but they still perform most hysterectomies through the not recommended abdominal route. There are several potential reasons for this behaviour. Firstly, abdominal hysterectomy is the traditional route of hysterectomy. Senior gynecologists have performed hysterectomies through this route for years and they probably feel comfortable with this technique. The majority seems not willing to alter their praxis despite better knowledge. 

Secondly, lack of laparoscopic competence by senior gynecologists prevents trainees from learning minimal invasive procedures that over time may cause this unfavourable trend to be continued. 

Thirdly, training courses in minimal invasive procedures are not that well established by the gynecological associations in many countries. It is consequently difficult and requires personal engagement as well as support from the hospitals and departments to achieve the needed competence to alter surgical praxis. Therefore, it takes dedicated gynecologists to make local changes. 

Reich et al. described the first laparoscopic hysterectomy decades ago [5]. Since then, the laparoscopic approach for hysterectomy has been adopted in many university hospitals and minimal invasive units. However, the rate of abdominal hysterectomy remains high, indicating that the general adaption of laparoscopic hysterectomy among gynecologists is still disappointingly low. Our study shows that change towards a laparoscopic approach even in smaller county hospitals is possible; however it requires dedicated gynecologists who want to change the current practice. When laparoscopic supracervical hysterectomy was introduced in 2003, the department possessed the knowledge to perform minimal invasive hysterectomies, but that is probably not sufficient if proper dedication is missing. Generally, it could be assumed that it would be more difficult to introduce TLH rather than LSH, because TLH is a more advanced procedure that requires laparoscopic suture skills. But, as shown in our study, the complexity of the procedure was not the preventive issue counteracting further increase in the percentage of laparoscopic hysterectomies. 

It could be argued why not vaginal hysterectomy was established as the preferred route of hysterectomy in place of laparoscopic hysterectomy. As the Cochrane review recommends, vaginal hysterectomy is the route of choice. On the other hand, if the complete field of gynecological surgery is evaluated, a large quantity of the operations could be performed laparoscopically. Increasing laparoscopic skills by performing supracervical and total hysterectomies may contribute to further development of surgical competence. Thereby, the majority of the gynecological procedures could be performed minimally invasive. 

Effort should be made to reduce the percentage of hysterectomies performed abdominally as the patients will benefit from a shift towards a less invasive procedure.

## 5. Conclusion

It is possible for a county hospital to alter their praxis and perform mini invasive hysterectomies, but it requires dedicated gynecologists. This change to an advanced procedure like total laparoscopic hysterectomy could be achieved without patients suffering from major complications. 

## Figures and Tables

**Table 1:** Patient's characteristics.

	Total laparoscopic hysterectomy *n* = 58	Percentage %
Age, years		
Mean ± SD	51.2 ± 13.0	
BMI		
Mean ± SD	27.0 ± 6.4	
Parity		
0	7	12.1
≥1	51	87.9
Previous cesarean section, no. (%)	10	17.2
Previous conisation, no. (%)	12	20.7
Adhesions present, no. (%)	5	8.6
Indication for the operation		
Menorrhagia/leiomyoma	25	43.1
Endometrial cancer, FIGO stage 1	19	32.6
Adenocarcinoma in cervix uteri	2	3.4
Previous conisation, CIN III	4	6.9
Dysmenorrhoea	2	3.4
Adenomyosis	1	1.7
Hereditary cancer risk	3	5.2
Pyometra	1	1.7
Hyperplasia	1	1.7
